# Posttuberculosis Consequences on Tuberculosis Prevention Effectiveness and Cost-effectiveness among New Immigrants, Canada 

**DOI:** 10.3201/eid3209.260473

**Published:** 2026-09

**Authors:** Aotikuer Ainiwaer, Aashna Uppal, Kevin Schwartzman, Kamila Romanowski, Sarah K. Brode, James C. Johnston, Jonathon R. Campbell

**Affiliations:** McGill University, Montreal, Quebec, Canada (A. Ainiwaer, K. Schwartzman, J.R. Campbell); University of Oxford, Oxford, UK (A. Uppal); Research Institute of the McGill University Health Centre, Montreal (K. Schwartzman, J.R. Campbell); McGill International TB Centre, Montreal (K. Schwartzman, J.R. Campbell); University of British Columbia, Vancouver, British Columbia, Canada (K. Romanowski, J.C. Johnston); British Columbia Centre for Disease Control, Vancouver (K. Romanowski, J.C. Johnston); University of Toronto, Toronto, Ontario, Canada (S.K. Brode); University Health Network, Toronto (S.K. Brode)

**Keywords:** *Mycobacterium tuberculosis*, tuberculosis and other mycobacteria, bacteria, respiratory infections, modelling, infectious disease, prevention, public health, Canada

## Abstract

Cost-effectiveness analyses of tuberculosis (TB) preventive treatment rarely consider long-term TB consequences. We examined how long-term TB consequences influence the effectiveness and cost-effectiveness of TB prevention among persons who immigrate to Canada. By using a Markov microsimulation model of 400,000 persons immigrating to Canada in 2025, we compared current postarrival TB infection screening levels (0.5%) with a screening level of 68% among new immigrants. Modelling scenarios included acute TB consequences and long-term TB-related death, illness, and healthcare costs (25-year horizon, 1.5% discount rate). Including long-term TB-related consequences increased the estimated TB-related quality-adjusted life years (QALYs) lost by 2.1-fold (95% uncertainty range 1.7–2.5-fold). When long-term TB-related consequences were included, estimated QALYs gained from expanded screening increased 2.4-fold (95% uncertainty range 1.6–4.4-fold), reducing the cost per QALY from $235,088 to $100,742 CAD. Ignoring long-term TB consequences substantially underestimates the effectiveness and cost-effectiveness of preventive interventions; such consequences should be incorporated into future evaluations.

Tuberculosis (TB) is the world’s leading cause of death from a single infectious agent, killing an estimated 1.23 million persons worldwide in 2024 (*1*). Shifts in global migration patterns have changed the epidemiologic landscape for TB in low-incidence regions, and migrant populations bear disproportionately high TB effects (*2*). In migrant populations, most TB disease occurs from the progression of TB infection acquired before immigration (*3*). Therefore, providing TB preventive treatment (TPT) to persons who recently immigrated should be considered among other TB elimination strategies (*4*). However, results of cost-effectiveness analyses evaluating systematic screening and treatment for TB infection among persons who recently immigrated to low-incidence settings have been mixed (*5*,*6*), and the implementation of such programs is rare (*7*). Also, there is substantial variability in how existing studies consider the risks and consequences of TB disease and TPT (*8*). Nearly all studies only consider the acute consequences (disease and death) of TB during the treatment period, omitting the long-term illness and death associated with post-TB consequences (*9*). However, emerging research has highlighted that healthcare utilization spikes in the years after TB treatment (*10*), and TB survivors are at substantially higher risk for illness and death compared with those who never had TB (*11*,*12*).

Failure to account for the long-term consequences of TB disease might underestimate the quality-adjusted life years (QALYs) gained and cost-effectiveness of TB infection screening and TPT. A global analysis of disability-adjusted life years associated with TB suggests post-TB consequences account for half of the total TB-related health issues (*9*). Similarly, an analysis of a hypothetical preventive intervention in high-incidence, low-middle-income settings found considering post-TB consequences substantially reduced estimated incremental cost-effectiveness ratios (ICERs) (*13*). However, those analyses were only illustrative and focused primarily on low-middle-income settings. Understanding how post-TB consequences might inform public health decisions in a low-incidence, high-income setting would be useful.

We conducted a modeling analysis to evaluate how post-TB consequences might affect effectiveness and cost-effectiveness of TB prevention among persons who recently immigrated to Canada. We model how incorporating post-TB health consequences affects QALYs lost because of TB and the cost-effectiveness of TB prevention programs under different assumptions.

## Methods

### Setting and Model Overview

Canada is a low TB incidence country, reporting 2,508 persons with TB disease in 2024, corresponding to an incidence of 6.1/100,000 population (*14*). Overall, 83% of all persons with TB in Canada were born outside the country; most of those persons are now permanent residents and citizens of Canada (*15*). Currently, the Canada immigration medical exam focuses on detection of TB disease before entry into Canada. No provincial or territorial programs provide systematic screening and treatment for TB infection for all recently immigrated persons (*16*), and only 0.5% of persons with clinical risk factors for TB receive screening (*17*).

Because of that context, we developed a microsimulation model to evaluate the costs, effectiveness, and cost-effectiveness of a systematic postarrival TB infection screening program in persons who recently and permanently migrated to Canada. We adapted a Markov microsimulation model by using TreeAge Pro Healthcare 2025 (TreeAge Software, LLC, https://www.treeage.com) (A. Uppal et al., unpub. data). Canada immigration targets for 2025 were 395,000 persons (*18*). We rounded that target to 400,000 persons and simulated a hypothetical cohort structured according to the age and country of origin of new permanent residents of Canada in 2024 (*19*). We used a previous modeling study to estimate the prevalence of TB infection among persons who recently immigrated to Canada, according to age and TB incidence in country of origin (Appendix Table 1). We estimated the risk for progression to TB disease by using the Public Health Agency of Canada data detailing time to development of TB disease after immigration (Appendix) (*15*).

The model compares the existing level of TB infection screening and treatment among persons who recently immigrated to Canada (status quo) with a scaled-up TB infection screening and treatment program (intervention). In the intervention strategy, all new permanent residents whose country of origin has an annual TB incidence of >50/100,000 persons would be asked to participate in TB infection screening after arrival in Canada. If the person tested positive for TB infection, they would be given TPT. We assumed 68% of persons would comply with the request on the basis of adherence evidence from other postarrival follow-up activities among immigrants (*20*). In both status quo and intervention strategies, the tuberculin skin test is used for screening, and 4 months of daily rifampin is the TPT regimen (*21*). A simplified model structure, including health states, is provided (Appendix Figure 1).

The primary outcomes were QALYs, TB episodes, TB deaths, and TB-related costs accrued over the 25-year time horizon for both strategies. We took a TB services perspective for this analysis. All outcomes were discounted at a rate of 1.5% per annum, in line with recommendations from Canada (*22*).

### Model Parameters

Whenever possible, we used systematic reviews and, if unavailable, high quality randomized trials and data from Canada to source all model parameters and set their distributions for probabilistic analysis (Table 1; Appendix Table 2). Most costs associated with TB came from a pan-Canada costing study (*23*). All cost parameters are expressed in 2023 Canadian dollars; we used consumer price indices to inflate costs to this year.

**Table 1:** Key model inputs for immigration TB infection screening and treatment of permanent residents in Canada*

Parameter	Base-case estimate	PSA distribution (range)
Health utilities		
Health utility: healthy or TB infection	0.81	Beta (0.69–0.91)
Annual absolute QALY loss because of TB adverse event†	0.0077	PERT (0.000–0.0096)
Annual absolute QALY loss associated with TB disease‡	0.057	PERT (0.033–0.100)
Annual relative utility decrement because of post-TB illness	0.018	PERT (0.000–0.044)§
Costs, 2023 CAD		
TST screening	$38	Gamma ($22–$59)
Medical evaluation before TPT	$237	Gamma ($136–$367)
Complete TPT, 4R	$515	Gamma ($163–$1,066)
Incomplete TPT, 4R	$234	Gamma ($21–$687)
Contact investigation¶	$5,429	Gamma ($3,147–$8,266)
Complete TB disease treatment	$21,897	Gamma ($3,260–$57,909)
Incomplete TB disease treatment	$18,495	Gamma ($13,815–$31,810)
TB-related death	$30,562	Gamma ($8,432–$66,681)
Post-TB healthcare, 5 years	$3,383	Gamma ($1,934–$5,231)
Model probabilities and transition parameters		
TST sensitivity	0.78	Beta (0.74–0.82)
TST specificity	0.94	Beta (0.80–1.00)
Proportion of population with TB infection	Country- and age-specific#	PERT
TB infection screening uptake, status quo	0.005	Beta (0.003–0.010)
TB infection screening uptake, intervention	0.68	Beta (0.64–0.72)
Returning for TST reading	0.94	Beta (0.90–0.98)
Initiating TPT	0.88	Beta (0.80–0.94)
Completing TPT, 4R	0.73	Beta (0.65–0.81)
Effectiveness of complete TPT	0.90	Beta (0.63–1.00)
Annual progression from TB infection to active TB per 1,000 person-years	0.85	lognormal (0.75–1.0)
Serious adverse event with TPT, 4R, <50 y	0.0068	PERT (0.0039–0.0097)
Serious adverse event with TPT, 4R, >50 y	0.013	PERT (0.005–0.021)
Death during TPT, 4R	0.00002	Beta (0.00000–0.000074)
Recurrence after TB treatment, annual probability during first 2 y	0.015	Triangular (0.0075–0.025)
Death hazard ratio after TB treatment	1.69	PERT (1.50–1.91)
TB-related case fatality probability, age group, y		
0–14	0.009	Fixed
15–24	0.004	Fixed
25–64	0.014	Fixed
>65	0.056	Fixed
Proportions of persons who immigrate to Canada**		
Age group, y**		
0–14	0.288	Fixed
15–34	0.385	Fixed
35–54	0.291	Fixed
55–74	0.036	Fixed
>75	0.001	Fixed
TB incidence in country of origin, cases/100,000 persons		
0–9	0.151	Fixed
10–49	0.186	Fixed
50–99	0.215	Fixed
100–199	0.241	Fixed
>200	0.208	Fixed

*4R, 4 mo of rifampin; PERT, project evaluation and review technique; PSA, probabilistic sensitivity analysis; QALY, quality-adjusted life years; TB, tuberculosis; TPT, TB preventive treatment; TST, tuberculin skin test; UR, uncertainty range. †Utility decrement of 0.2 for 2 weeks. ‡Health utility decrement of 0.17 for 4 mo. §From Nicolas Menzies (see Acknowledgments); in the communication the post-TB disability weight used for Canada was 0.018 (0.005–0.044). We further reduced the lower bound of the uncertainty interval from 0.005 to 0 to enable evaluation of no disease occurrence. ¶Cost includes nurse time for a contact investigation involving 4 contacts ($3,630) plus costs of evaluation, diagnosis, and treatment for TB infection and disease among contacts, under the assumption all were evaluated for TB disease and infection with 3% having disease and 33% having infection. #Appendix Table 1, https://wwwnc.cdc.gov/EID/article/32/9/26-0473-App1.pdf. **Numbers might not sum to 1 because of rounding.

We parameterized QALYs lost during acute TB consequences by using an estimated annualized loss of 0.057 (excluding death) (*24*) and age-specific risk for death during acute TB by using surveillance data from Canada (*15*). We categorized post-TB consequences as increased risk for death after successful TB treatment, persistent illness because of long-term disability, and excess healthcare costs after TB (*11*). We assumed the risk for death after TB treatment was 69% (95% CI 50%–91%) higher than among age-matched persons without a history of TB disease (*25*) and that the increased risk persisted for life. We parameterized background risk of death from TB according to lifetables from Canada (*26*). In addition, we permanently reduced all TB survivors’ health utility by 1.8% (95% CI 0%–4.4%) (*28*). Finally, we estimated TB survivors would have excess healthcare costs of $3,383 (95% CI $1,934–$5,231) over 5 years after successful TB treatment (*10*), evenly distributed across each of the 5 years. We modeled each of those 3 post-TB consequences separately and together.

### Analysis

Our Markov microsimulation model sampled from the associated distribution of each parameter 2,000 times to generate 2,000 parameter sets. For each parameter set, we simulated 400,000 persons and then estimated mean outcomes and 95% uncertainty ranges by using the 2.5th and 97.5th percentiles.

We projected outcomes for 5 scenarios. Each scenario included acute TB consequences: no post-TB consequences, post-TB death only, post-TB illness only, excess post-TB healthcare costs only, and all 3 post-TB consequences.

To estimate the effect of post-TB consequences on the estimated QALYs lost because of TB, we quantified QALYs lost because of TB under the status quo for each post-TB scenario. Next, we calculated incremental costs, QALYs, TB episodes, and TB deaths for the intervention strategy compared with the status quo for each scenario. We calculated the incremental cost per QALY gained and estimated the proportion of simulations where ICERs fell below several willingness-to-pay thresholds (WTP) per QALY, including $50,000, $100,000, and $150,000.

To further understand the relative importance of post-TB consequences to outcomes of cost-effectiveness, we performed a partial rank correlation coefficient (PRCC) analysis examining the different model parameters. For that analysis, the outcome was the incremental net monetary benefit (iNMB) of the intervention compared with the status quo for the scenario where all 3 post-TB consequences are considered calculated by using a WTP threshold of $100,000 per QALY. We calculated the iNMB by multiplying the incremental QALYs by the WTP and subtracting the incremental intervention costs.

To assess sensitivity of results to post-TB consequence parameters, we selected the 200 simulations with the lowest (0–10th percentile) and highest (90th–100th percentile) values for each parameter (increased disease, death, and healthcare costs). For each of those simulation sets, we estimated incremental outcomes, the ICER, and the proportion with ICERs below a WTP of $100,000 per QALY.

We performed 5 sets of scenario analyses. First, we increased our estimates of the QALYs lost because of the acute consequences of TB to 0.11 (parameterized with a minimum value of 0.03 and maximum of 0.2) (*27*). Second, in place of a tuberculin skin test, we modeled the use of an interferon-γ release assay, which had identical sensitivity but improved specificity (*28*), 100% test completion, and higher cost ($59) (*23*). We parameterized specificity by using a β distribution (α = 90 and β = 4) and the cost with a gamma distribution (shape = 60 and scale = 0.98). Third, we changed the annual discount rate to 0% and 3% (*22*). Fourth, we made our post-TB death parameter more conservative (9% increase, 95% uncertainty range [UR] 2%–17%), which we approximated on the basis of excess death expected from reduced lung function after TB (*9*). Fifth, we evaluated the effect of a lower rate of TB progression (0.75 [95% UR 0.6–0.9]/1,000 person years) on outcomes (*29*).

## Results

Under the status quo and considering only acute TB consequences, we projected 927 (95% UR 764–1,123) persons to develop TB and 30 (95% UR 24–36) to die from TB; we projected 312 (95% UR 241–386) QALYs lost. We projected TB-related health system expenditures to total $25.3 million (95% UR $7.6–$61.9 million) over the 25-year time horizon (Table 2; Appendix Table 3). Inclusion of post-TB death increased the estimated number of QALYs lost because of TB 1.6-fold (95% UR 1.4–1.7-fold), representing a 60% increase compared with acute TB consequences only. Similarly, inclusion of post-TB illness increased the estimated number of QALYs lost because of TB by 1.5-fold (95% UR 1.2–2.0-fold). Although including excess healthcare costs post-TB did not affect QALYs lost, it increased estimated TB-related health system expenditures to $27.6 million (95% UR $9.7–$64.5 million). When all post-TB consequences were included, TB was projected to result in 657 (95% UR 479–876) QALYs lost, a 2.1-fold (95% UR 1.7–2.5-fold) increase compared with estimates excluding post-TB consequences (Table 2; Appendix Table 3). This result suggests post-TB consequences accounted for 52% (95% UR 41%–60%) of QALYs lost because of TB.

**Table 2:** Total quality adjusted life years lost because of TB when considering acute and long-term consequences in the status quo scenario*

QALY loss	Acute TB consequences only, referent	Post-TB elevated risk for death	Post-TB persistent disease	Elevated risk for death and persistent disease
Absolute QALY loss because of TB	312 (241–386)	491 (375–619)	482 (338–665)	657 (479–876)
Relative QALY loss because of TB compared with no post-TB consequences	1.0	1.6 (1.4–1.7)	1.5 (1.2–2.0)	2.1 (1.7–2.5)

*Values are no. (95% uncertainty range). TB, tuberculosis; QALY, quality adjusted life year.

Across all scenarios, a postarrival TB infection screening intervention was estimated to result in QALY gains, increased costs, and reduced TB episodes and deaths (Table 3). Compared with a scenario including only acute TB consequences, including post-TB death resulted in a slightly greater increase in estimated incremental QALYs gained by the intervention than including post-TB illness. When all 3 post-TB consequences were incorporated, estimated QALY gains increased by 2.4-fold (95% UR 1.6–4.4-fold), estimated TB deaths averted increased by 2.2-fold (95% UR 1.6–3.0-fold), and incremental costs were projected to be 4% (95% UR 1%–13%) lower compared with the exclusion of post-TB consequences.

**Table 3:** Effects of post-tuberculosis consequences on incremental outcomes between intervention and status quo*

Incremental outcomes between status quo and intervention	Acute TB consequences only, referent	Post-TB elevated risk for death	Post-TB persistent disease	Post-TB healthcare costs	All post-TB consequences
Absolute incremental cost, millions, 2023 CAD	19.4 (4.1–38.0)	19.4 (4.1–38.0)	19.4 (4.1–38.0)	18.7 (3.3–37.3)	18.7 (3.3–37.3)
Relative incremental cost compared with no post-TB consequences	1.0	1.0	1.0	0.96 (0.87–0.99)	0.96 (0.87–0.99)
Absolute incremental QALY	82 (28–124)	138 (76–198)	131 (65–200)	82 (28–124)	186 (109–267)
Relative incremental QALY ratio compared with no post-TB consequences	1.0	1.7 (1.3–3.0)	1.7 (1.2–2.7)	1.0	2.4 (1.6–4.4)
Absolute incremental TB-related deaths	−12 (−15 to −7)	−25 (−33 to −18)	−12 (−15 to −7)	−12 (−15 to −7)	−25 (−33 to −18)
Relative incremental TB deaths ratio compared with no post-TB consequences	1.0	2.2 (1.6–3.0)	1.0	1.0	2.2 (1.6–3.0)
ICER	$235,088	$140,146	$147,595	$227,340	$100,742
<$50,000, %	3	5	5	3	11
<$100,000, %	8	27	25	9	53
<$150,000, %	21	59	54	24	81

*Incremental outcomes refer to the difference between intervention and status quo per 400,000 persons. Values are (95% uncertainty range). ICER, incremental cost-effectiveness ratios; QALY, quality adjusted life year; TB, tuberculosis.

When only acute consequences of TB were included, the estimated ICER of a postarrival TB infection screening intervention was $235,088 per QALY gained, compared with the status quo. When all 3 post-TB consequences were included, the ICER was reduced to $100,742. In that scenario, 53% of simulations had an ICER below a WTP threshold of $100,000, compared with only 8% when only acute consequences were included (Table 3; Appendix Figure 2).

When all post-TB consequences are included, the PRCC analysis showed that post-TB illness and death were positively correlated with iNMB, although excess post-TB healthcare costs were not (Figure). TB infection test specificity, cost of TB disease treatment, TPT effectiveness, annual TB progression rates, and health disutility from post-TB illness were the 5 parameters most positively correlated with iNMB. Post-TB death was the 11th most positively correlated parameter with iNMB. Similarly, we found estimates of effectiveness and cost-effectiveness of postarrival TB infection screening were most sensitive to the disutility associated with post-TB illness and minimally sensitive to post-TB death and excess post-TB healthcare costs (Appendix Table 4).

**Figure:**
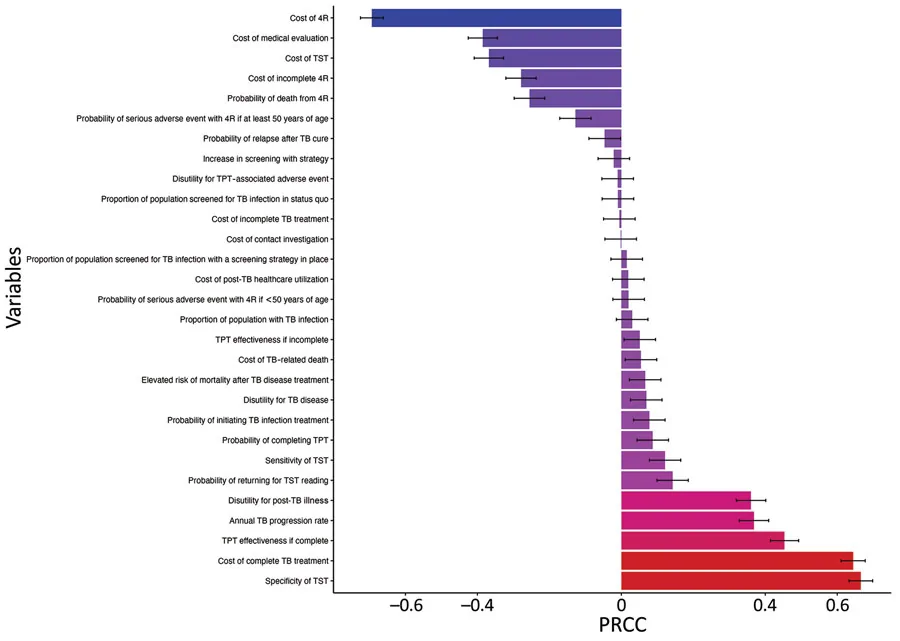
PRCC for incremental net monetary benefit as drivers of cost-effectiveness of tuberculosis prevention among immigrants, Canada. Incremental net monetary benefit refers to the difference in net monetary benefit between the intervention and the status quo. In this analysis, parameters with more extreme positive PRCC estimates improve the incremental net monetary benefit as they increase. Parameters with more extreme negative PRCC estimates worsen the incremental net monetary benefit as they increase. Error bars indicate 95% uncertainty range. 4R, 4 months of rifampin treatment; PRCC, partial rank correlation coefficient; TB, tuberculosis; TPT, tuberculosis preventive treatment; TST, tuberculin skin test.

Estimates of the relative contribution of post-TB consequences to QALYs lost because of TB disease were inversely correlated to the acute consequences of TB (Appendix Table 5), but overall ICERs were more favorable when post-TB consequences were included (Appendix Table 6). Substitution of an interferon-γ release assay for the tuberculin skin test did not substantially affect estimated ICERs (Appendix Table 7) because specificity gains were offset by increased costs. Relative increases in QALYs gained after incorporating post-TB consequences and PRCC estimates were largely unchanged across discount rates (Appendix Tables 8, 9, Figures 3, 4). Unsurprisingly, ICERs were more favorable with no discounting and more unfavorable with 3% discounting. When all post-TB consequences were included, the estimated ICER was $71,714 per QALY gained, and 76% of simulations were <$100,000 per QALY gained with no discounting, whereas the corresponding outcomes with 3% discounting were $137,407 and 27%.

A more conservative parameterization of post-TB death substantially reduced the effect on QALYs lost because of TB and QALYs gained by a screening program. With a more conservative parameterization (9% vs. 69%), including post-TB death only increased the number of QALYs lost because of TB by 5% (95% UR 2%–9%). As a result, overall post-TB consequences accounted for a smaller proportion of QALYs lost because of TB than our base analysis at 44% (95% UR 23%–64%). In addition, the more conservative post-TB death risk reduced its influence on QALYs gained through the screening program and on the ICER (Appendix Tables 10, 11). Reducing the TB progression rate did not affect long-term TB consequence effect on QALYs gained through a screening program, but it did reduce the estimated cost-effectiveness (Appendix Tables 12, 13).

## Discussion

We found excluding post-TB consequences of illness, death, and excess healthcare costs can lead to substantially undervaluing TB prevention strategies in a low-incidence, high-income setting like Canada. Post-TB consequences account for a large fraction of QALYs lost because of TB. Including post-TB consequences in our analysis of a post-arrival TB infection screening and preventive treatment intervention among persons immigrating to Canada more than doubled the estimated QALYs gained and reduced the estimated ICER by >50%.

One of the most studied use cases for TB infection screening and preventive treatment in low-incidence countries is among persons who recently immigrated (*4*,*30*,*31*). Yet, the literature on that topic is largely divided when it comes to cost-effectiveness (*8*). Most of the literature evaluating cost-effectiveness has not incorporated post-TB consequences. A recent systematic review (*8*) found only 3 (*32*–*34*) of 14 included studies evaluating postarrival TB infection screening in immigrants considered post-TB consequences. None of those 3 studies considered increased healthcare cost, and only 1 study (*34*) considered elevated risk for death after TB. Although all 3 studies considered persistent illness after TB, its inclusion was highly variable across studies, which is unsurprising because of the paucity of prospective cohort data estimating health utility decrements associated with post-TB illness. Improving our understanding of the long-term effect of TB on health utility should be a priority, considering our finding that the cost-effectiveness estimates for TB prevention were sensitive to post-TB illness parameterization.

A global analysis of the health burden of incident TB estimated that 47% (95% UR 37%–57%) of TB-related disability-adjusted life years were attributed to post-TB illness and death (*9*). In our study, post-TB consequences accounted for 52% (95% UR 41%–60%) of TB-related QALYs lost. Our higher estimate might reflect a lower number of deaths and lower health disutility during the acute phase of TB in Canada but higher estimated post-TB death. When we used a conservative estimate of post-TB death, like the previous global study, our estimated contribution was only 44% (95% UR 23%–64%).

By neglecting the contribution of post-TB consequences on health disutility, most literature to date has likely undervalued the potential benefits of TB prevention strategies. Because ICERs for TB prevention among recent immigrants are typically above common WTP thresholds (*30*,*31*), reductions in the ICER might have meaningful policy implications. Also, ensuring that cost-effectiveness is accurately and comprehensively measured is necessary for policymakers seeking to prioritize scarce healthcare funds.

The first major strength of our study is that we parameterized post-TB consequences in our analysis by using high-quality data from Canada or from other published literature, with appropriate consideration of uncertainty. Second, we leveraged a common use case for our analysis, with our model structured to capture necessary elements of the care cascade and TB natural history. Finally, we also evaluated several implications of ignoring post-TB consequences relevant to both researchers and decision-makers.

The first limitation of our study is that we did not model secondary TB transmission beyond the cost of routine contact investigations. Including future TB cases resulting from secondary transmission would likely improve the estimated cost-effectiveness of the intervention. Second, we assumed that the observed increases in death, disease, and healthcare costs after TB were attributable to TB only and therefore preventable through TPT. Although we carefully adjusted the analyses of our underlying parameterization of those estimates, it is not possible to determine if TB was the only cause of those outcomes (*35*), and there might have been residual confounding factors. Third, we parameterized the elevated risk for illness and death post-TB to be lifelong. Our estimate of post-TB death risk was derived from a population-based study of permanent residents of British Columbia with a mean follow-up of 13 years. The elevated death risk persisted throughout the study period, suggesting it might be long-term. However, data on post-TB illness are less certain. Post-TB respiratory disease is heterogeneous (*35*), and some data suggest that the increase in post-TB respiratory disease might dissipate over time (*36*). However, post-TB illness is not limited to the respiratory system, and nonrespiratory disease might be substantial (*37*,*38*). Those 2 considerations might lead to inaccurately assessing the effect of post-TB illness. Finally, we only considered health system costs related to TB. We did not include costs incurred by patients and caregivers during or after treatment. No comprehensive data on those costs are available in Canada (*39*). It is unclear how including patient-incurred costs would affect cost-effectiveness because patients incur costs during both TB disease and TPT (*40*,*41*).

In summary, neglecting post-TB consequences underestimated the effectiveness of a postarrival infection screening and treatment intervention among persons who recently immigrated to Canada by more than 2-fold. Including all post-TB consequences reduced the estimated ICERs from $235,088 to $100,742 per QALY gained. Our findings suggest the long-term health and economic burden of TB substantially affects how TB prevention is valued. Those findings should motivate TB preventive efforts and prompt the continued collection of relevant cohort data to fill critical knowledge gaps. We encourage the consistent inclusion of post-TB consequences in analyses of TB prevention to guide policy in low-incidence settings moving forward.

Appendix Additional information about posttuberculosis consequences on tuberculosis prevention effectiveness and cost-effectiveness among new immigrants, Canada.
