# Supplementary material for: Posttuberculosis Consequences on Tuberculosis Prevention Effectiveness and Cost-effectiveness among New Immigrants, Canada
Source: Emerg Infect Dis. 2026 Sep;32(9):1421–9. doi: 10.3201/eid3209.260473 (PMC13555736; doi:10.3201/eid3209.260473)
Supplement: Supplementary file 1 [file 26-0473-Techapp-s1.pdf]

Article DOI: https://doi.org/10.3201/eid3209.260473

EID cannot ensure accessibility for supplementary materials supplied by authors. Readers who have difficulty accessing supplementary content should contact the authors for assistance

Posttuberculosis Consequences on Tuberculosis Prevention Effectiveness and Cost-effectiveness among New Immigrants, Canada

Appendix

Appendix Methods (Estimating Progression Rate)

To estimate the annual progression rate from tuberculosis (TB) infection to TB disease, we leveraged data reported by the Public Health Agency of Canada in their Expanded Report on Tuberculosis for the years 2012–2021, specifically Table 8 (https://www.canada.ca/en/public-health/services/publications/diseases-conditions/tuberculosis-canada-expanded-report-2012-2021.html#a1.8). This table details the number of people born outside of Canada who developed TB by time since arrival. The groups include <2y (arrived 2021 or 2020), 3–4 years (arrived 2019 or 2018), 5–6 years (arrived 2017 or 2016), 7–8 years (arrived 2015 or 2014), 9–10 years (arrived 2013 or 2012), and >10 years (which for the purposes of this estimation we assumed included people arriving between 1997 and 2011). For each year of arrival, we used Immigration, Refugees, and Citizenship Canada data on the number of new permanent residents to Canada to approximate the denominator for each category.

Based on the observed number of people developing tuberculosis in Canada who reported a birthplace outside of Canada, we estimated the number who were citizens or permanent residents based on the historical distribution of TB episodes among temporary versus permanent residents (75%); i.e., our denominator. Using the distribution of TB episodes reported by time since arrival reported in Table 8 of the report cited above, we estimated a numerator. We then estimated the annual TB risk based on this calculated numerator and our estimated denominator. Thus, for the year 2021, we could estimate the number of people developing TB based on time

since arrival for those arriving the past year up to those arriving 25 years prior. We used the distribution of TB risk to estimate a TB progression rate decline with time. Specifically, we observed in the first 6 years after arrival, annual TB risk was approximately similar (0.016%), which then fell in years 7–10 (0.013%), and again for years >10 (0.011%). This provided us with (*1*) an estimated number of people who would develop TB over a 25-year period and (*2*) a risk decay function we could embed in our model. Thus, we estimated TB risk would fall by 20% for years 7–10 after arrival when compared to years 1–6 and by 30% for years >10 when compared to years 1–6.

Using these data, in our model, we set a target for the total number of people developing TB over a 25-year period to be 1200 in a cohort of 400,000 new immigrants (undiscounted). The age and TB incidence in country of origin distribution is described in the main text (Table 1) and in this supplement (Table S1). We then varied the rate of TB progression against this overall TB target of 1200 and arrived at an annual progression rate from infection to disease of 1.1 per 1000 person years for years 1–6 (and subsequent declines in later years) to satisfy this target. We varied the parameter±0.2 per 1000 person years for the purposes of fitting a lognormal distribution for probabilistic analysis.

When this initial rate, and its subsequent decline in later years, is modeled, it is equivalent to an average annual progression rate of 0.85 (95% UR: 0.75 – 1.0) per 1000 person years over the 25-year time horizon. In sensitivity analysis, we evaluated a lower rate based on a different immigration cohort year, which was equal to 0.75 (95% UR: 0.6 – 0.9) per 1000 person years over the 25-year time horizon.

References

1. Bauer M, Ahmed S, Benedetti A, Greenaway C, Lalli M, Leavens A, et al. Health-related quality of life and tuberculosis: a longitudinal cohort study. Health Qual Life Outcomes. 2015;13:65. PubMed https://doi.org/10.1186/s12955-015-0250-4

2. McLernon DJ, Dillon J, Donnan PT. Health-state utilities in liver disease: a systematic review. Med Decis Making. 2008;28:582–92. PubMed https://doi.org/10.1177/0272989X08315240

3. Menzies NA, Quaife M, Allwood BW, Byrne AL, Coussens AK, Harries AD, et al. Lifetime burden of disease due to incident tuberculosis: a global reappraisal including post-tuberculosis sequelae. Lancet Glob Health. 2021;9:e1679–87. PubMed https://doi.org/10.1016/S2214-109X(21)00367-3

4. Campbell JR, Nsengiyumva P, Chiang LY, Jamieson F, Khadawardi H, Mah HK, et al. Costs of tuberculosis at 3 treatment centers, Canada, 2010–2016. Emerg Infect Dis. 2022;28:1814–23. PubMed https://doi.org/10.3201/eid2809.220092

5. Fassbender K, Fainsinger RL, Carson M, Finegan BA. Cost trajectories at the end of life: the Canadian experience. J Pain Symptom Manage. 2009;38:75–80. PubMed https://doi.org/10.1016/j.jpainsymman.2009.04.007

6. Romanowski K, Law MR, Karim ME, Campbell JR, Hossain MB, Gilbert M, et al. Healthcare utilization after respiratory tuberculosis: a controlled interrupted time series analysis. Clin Infect Dis. 2023;77:883–91. PubMed https://doi.org/10.1093/cid/ciad290

7. Canadian Institute for Health Information. Patient cost estimator, [cited 2025 Nov 28]. https://www.cihi.ca/en/patient-cost-estimator

8. Campbell JR, Pease C, Daley P, Pai M, Menzies D. Diagnosis of tuberculosis infection. Can J Respir Crit Care Sleep Med. 2022;6(S1):49–65. https://doi.org/10.1080/24745332.2022.2036503

9. Zwerling A, Behr MA, Verma A, Brewer TF, Menzies D, Pai M. The BCG World Atlas: a database of global BCG vaccination policies and practices. PLoS Med. 2011;8:e1001012. PubMed https://doi.org/10.1371/journal.pmed.1001012

10. Jordan AE, Nsengiyumva NP, Houben RMGJ, Dodd PJ, Dale KD, Trauer JM, et al. The prevalence of tuberculosis infection among foreign-born Canadians: a modelling study. CMAJ. 2023;195:E1651–9. PubMed https://doi.org/10.1503/cmaj.230228

11. Immigration Refugees Citizenship Canada. Canada - Permanent Residents by Province/Territory and Immigration Category, 2017 [cited 2025 Nov 28] https://open.canada.ca/data/en/dataset/f7e5498e-0ad8-4417-85c9-

12. Statistics Canada. Life expectancy at birth and at age 65, by province and territory, three-year average, 2017 [cited 2025 Nov 28]. https://www150.statcan.gc.ca/t1/tbl1/en/tv.action?pid=1310040901

13. Greenaway C, Diefenbach-Elstob T, Schwartzman K, Cook VJ, Giovinazzo G, Njoo H, et al. Tuberculosis surveillance and tuberculosis infection testing and treatment in migrants. Can J Respir Crit Care Sleep Med. 2022;6(sup1):194–204.

14. Campbell JR, Johnston JC, Cook VJ, Sadatsafavi M, Elwood RK, Marra F. Cost-effectiveness of latent tuberculosis infection screening before immigration to low-incidence countries. Emerg Infect Dis. 2019;25:661–71. PubMed https://doi.org/10.3201/eid2504.171630

15. Chan IHY, Kaushik N, Dobler CC. Post-migration follow-up of migrants identified to be at increased risk of developing tuberculosis at pre-migration screening: a systematic review and meta-analysis. Lancet Infect Dis. 2017;17:770–9. PubMed https://doi.org/10.1016/S1473-3099(17)30194-9

16. Alsdurf H, Hill PC, Matteelli A, Getahun H, Menzies D. The cascade of care in diagnosis and treatment of latent tuberculosis infection: a systematic review and meta-analysis. Lancet Infect Dis. 2016;16:1269–78. PubMed https://doi.org/10.1016/S1473-3099(16)30216-X

17. Winters N, Belknap R, Benedetti A, Borisov A, Campbell JR, Chaisson RE, et al. Completion, safety, and efficacy of tuberculosis preventive treatment regimens containing rifampicin or rifapentine: an individual patient data network meta-analysis. Lancet Respir Med. 2023;11:782–90. PubMed https://doi.org/10.1016/S2213-2600(23)00096-6

18. Menzies D, Adjobimey M, Ruslami R, Trajman A, Sow O, Kim H, et al. Four months of rifampin or nine months of isoniazid for latent tuberculosis in adults. N Engl J Med. 2018;379:440–53. PubMed https://doi.org/10.1056/NEJMoa1714283

19. International Union Against Tuberculosis Committee on Prophylaxis. Efficacy of various durations of isoniazid preventive therapy for tuberculosis: five years of follow-up in the IUAT trial. Bull World Health Organ. 1982;60:555–64. PubMed

20. Public Health Agency of Canada. tuberculosis in Canada: 2012–2021 expanded report, 2024 [cited 2025 Nov 28]. https://www.canada.ca/en/public-health/services/publications/diseases-conditions/tuberculosis-canada-expanded-report-2012-2021

21. Tiemersma EW, van der Werf MJ, Borgdorff MW, Williams BG, Nagelkerke NJ. Natural history of tuberculosis: duration and fatality of untreated pulmonary tuberculosis in HIV negative patients: a systematic review. PLoS One. 2011;6:e17601. PubMed https://doi.org/10.1371/journal.pone.0017601

22. Basham CA, Karim ME, Cook VJ, Patrick DM, Johnston JC. Post-tuberculosis mortality risk among immigrants to British Columbia, Canada, 1985-2015: a time-dependent Cox regression analysis of linked immigration, public health, and vital statistics data. Can J Public Health. 2021;112:132–41. PubMed https://doi.org/10.17269/s41997-020-00345-y

23. Immigration Refugees and Citizenship Canada. Permanent residents—monthly IRCC updates, 2025 [cited 2025 Nov 28]. https://open.canada.ca/data/en/dataset/f7e5498e-0ad8-4417-85c9-9b8aff9b9eda

Appendix Table 1. Probability of TB infection among immigrants.

Age group (years)	Estimate*	Values used as low and high parameters in PERT distribution
TB Incidence Band: 0 to 9 per 100,000		
0–14	0.005	(0.0025 - 0.01)
15–34	0.0225	(0.02 - 0.03)
35–54	0.05	(0.0375 - 0.0675)
55–74	0.07	(0.0625 - 0.08)
≥75	0.085	(0.075 - 0.095)
TB Incidence Band: 10 to 49 per 100,000		
0–14	0.0134	(0.0067 - 0.0268)
15–34	0.0603	(0.0536 - 0.0804)
35–54	0.134	(0.1005 - 0.1809)
55–74	0.1876	(0.1675 - 0.2144)
≥75	0.2278	(0.201 - 0.2546)
TB Incidence Band: 50 to 99 per 100,000		
0–14	0.019	(0.0905 - 0.038)
15–34	0.0855	(0.076 - 0.114)
35–54	0.19	(0.1425 - 0.2565)
55–74	0.266	(0.2375 - 0.304)
≥75	0.323	(0.285 - 0.361)
TB Incidence Band: 100 to 199 per 100,000		
0–14	0.0288	(0.0144 - 0.0576)
15–34	0.1296	(0.1152 - 0.1728)
35–54	0.288	(0.216 - 0.3888)
55–74	0.4032	(0.36 - 0.4608)
≥75	0.4896	(0.432 - 0.5472)
TB Incidence Band: ≥200 per 100,000		
0–14	0.0338	(0.0169 - 0.0676)
15–34	0.1521	(0.1352 - 0.2028)
35–54	0.338	(0.2535 - 0.4563)
55–74	0.4732	(0.4225 - 0.5408)
≥75	0.5746	(0.507 - 0.6422)

*Estimates from Jordan AE, Nsengiyumva NP, et al (*11*)
Abbreviations: TB = Tuberculosis

Appendix Table 2. Key model inputs for tuberculosis infection screening and treatment of permanent residents in Canada, including references.

Description	Estimate	Range for Probabilistic Sensitivity Analysis	Distribution Type	Reference
Health Utilities				
Health utility for healthy individual or individual with TB infection	0.81	(0.69 - 0.91)	Beta	(*1*)
Annual absolute QALY loss associated with adverse event due to TB infection treatment (utility decrement of 0.2 for 2 weeks)	0.0077	(0.00 - 0.0096)	PERT	Assumption, adjusted from (*2*)
Annual absolute QALY loss associated with TB disease (health utility decrement of 0.17 for 4 mo)	0.057	(0.033 - 0.1)	PERT	(*1*)
Relative reduction in health utility associated with post-TB morbidity	0.018	(0 - 0.044)	PERT‡	(*3*), personal communication
Costs (2023 $CAD)				
Cost of TB infection screening, TST	$38	($22 - $59)	Gamma	(*4*)
Cost of medical evaluation before TPT	$237	($136 - $367)	Gamma	(*4*)
Cost of TPT, complete 4R	$515	($163 - $1,066)	Gamma	(*4*)
Cost of TPT, incomplete 4R	$234	($21 - $687)	Gamma	(*4*)
Cost of contact investigation†	$5,429	($3,147 - $8,266)	Gamma	(*4*)
Cost of complete TB disease treatment	$21,897	($3,260 - $57,909)	Gamma	(*4*)
Cost of incomplete TB disease treatment	$18,495	($13,815 - $31,810)	Gamma	(*4*)
Cost of TB-related death	$30,562	($8,432 - $66,681)	Gamma	(*5*)
Cost of post-TB healthcare utilization, total over 5 y	$3,383	($1,934 - $5,231)	Gamma	(*6,7*)
Model Probabilities and Transitions				
Sensitivity of TST screening	0.78	(0.74 - 0.82)	Beta	(8)
Specificity of TST screening	0.941	(0.80 - 1.00)	Beta	(8,*9*)

Description	Estimate	Range for Probabilistic Sensitivity Analysis	Distribution Type	Reference
Proportion of population with TB infection	Depends on Country of Origin and Age (See Table S1)	Depends on Country of Origin and Age (See Table S1)	PERT	(*10*–*12*)
Proportion of population that is screened for TB infection in the status quo	0.005	(0.003 - 0.010)	Beta	(*13*)
Proportion of population that is screened for TB infection in the intervention	0.68	(0.64 - 0.72)	Beta	(*14,15*)
Probability of returning for TST reading	0.94	(0.90 - 0.98)	Beta	(*16*)
Probability of initiating TPT	0.88	(0.80 - 0.94)	Beta	(*16*)
Probability of completing TPT, 4R	0.73	(0.65 - 0.81)	Beta	(*17*)
Effectiveness of complete TPT	0.90	(0.63 - 1.00)	Beta	(*18*,*19*)
Average annual TB progression rate among people with TB infection	0.85 per 1000 person-years	(0.75 to 1.0)	Lognormal	Estimated (*20*)
Probability of serious adverse event with TPT, 4R	0.0068 if age <50, otherwise 0.013	(0.0039 - 0.0097) if age <50, otherwise (0.005 - 0.021)	PERT	(*17*)
Probability of death during TPT, 4R	0.00002	(0.00 - 0.000074)	Beta	(*17*)
Probability of TB-related death				
Age 0–14 y	0.009	-	Fixed	(*20*)
Age 15–24 y	0.004			
Age 25–64 y	0.014			
Age ≥65 y	0.056			
Probability of recurrence in each of the 2 y after completing TB disease treatment	0.015	(0.0075 – 0.025)	Triangular	(*21*)
Elevated risk of mortality after TB disease treatment	1.69	(1.50 - 1.91)	PERT	(*22*)
Age and TB incidence in country-of-origin distribution for people migrating to Canada				
Age distribution*				
Age 0–14 y	0.288	-	Fixed	For the year 2024 (*23*),
Age 15–34 y	0.385			
Age 35–54 y	0.291			
Age 55–74 y	0.036			
Age ≥75 y	0.001			
Annual TB incidence in country-of-origin distribution*				
0–9 per 100,000	0.151	-	Fixed	For the year 2024 (*23*),
10–49 per 100,000	0.186			
50–99 per 100,000	0.215			
100–199 per 100,000	0.241			
≥200 per 100,000	0.208			

*Numbers may not sum to 1 due to rounding.

‡From Nicolas Menzies, see acknowledgments; in the communication the post-TB morbidity disability weight used for Canada was 0.018 (0.005 to 0.044). To be conservative, we further reduced the lower bound of the uncertainty interval from 0.005 to 0 to still allow for no morbidity to occur.

†Cost includes nurse time for a contact investigation involving 4 contacts ($3,630) plus costs of evaluation, diagnosis, and treatment of tuberculosis infection and disease among contacts, under the assumption all were evaluated for tuberculosis disease and infection with 3% having disease and 33% having infection.

Abbreviations: TST = tuberculin skin test, TB = tuberculosis, 4R = 4 mo of rifampin, TPT = tuberculosis preventive treatment, QALY = quality-adjusted life years

Appendix Table 3. Detailed primary outcomes with each post-TB consequence

Strategy	How much does the strategy cost ($)? (95% UR)	How many QALYs does the strategy incur? (95% UR)	How many TB disease episodes does the strategy produce? (95% UR)	How many TB-related deaths does the strategy incur? (95% UR)
Status Quo without Post-TB Consequence	25.3 million (7.4 to 61.9 million)	6,630,976 (5,639,652 to 7,456,485)	927 (764 to 1,123)	30 (24 to 36)
Status Quo with Post-TB Mortality	25.3 million (7.4 to 61.9 million)	6,630,797 (5,639,542 to 7,456,242)	927 (764 to 1,123)	74 (59 to 92)
Status Quo with Post-TB Morbidity	25.3 million (7.4 to 61.9 million)	6,630,806 (5,639,580 to 7,456,300)	927 (764 to 1,123)	30 (24 to 36)
Status Quo with Post-TB Cost	27.6 million (9.7 to 64.5 million)	6,630,976 (5,639,652 to 7,456,485)	927 (764 to 1,123)	30 (24 to 36)
Status Quo with All Post-TB Consequences	27.6 million (9.7 to 64.5 million)	6,630,631 (5,639,470 to 7,456,062)	927 (764 to 1,123)	74 (59 to 92)
Intervention without Post-TB Consequence	44.7 million (26.2 to 77.0 million)	6,631,058 (5,639,768 to 7,456,628)	666 (540 to 817)	18 (14 to 25)
Intervention with Post-TB Mortality	44.7 million (26.2 to 77.0 million)	6,630,935 (5,639,656 to 7,456,449)	666 (540 to 817)	49 (36 to 65)
Intervention with Post-TB Morbidity	44.7 million (26.2 to 77.0 million)	6,630,937 (5,639,716 to 7,456,504)	666 (540 to 817)	18 (14 to 25)
Intervention with Post-TB Cost	46.3 million (27.9 to 78.5 million)	6,631,058 (5,639,768 to 7,456,628)	666 (540 to 817)	18 (14 to 25)
Intervention with All Post-TB Consequences	46.3 million (27.9 to 78.5 million)	6,630,816 (5,639,605 to 7,456,328)	666 (540 to 817)	49 (36 to 65)

Abbreviations: UR = uncertainty range, TST = tuberculin skin test, TB = tuberculosis, 4R = 4 mo of rifampin, Status Quo = 0.5% of permanent residents screened, all receiving TST for screening and 4R for treatment, Intervention = 68% uptake among permanent residents screened, all receiving TST for screening and 4R for treatment

Appendix Table 4. Estimated incremental outcomes among simulations with various post-tuberculosis parameters falling below the 10th or above the 90th percentile for their parameterized value.
In this analysis we order the parameter estimates from smallest to largest and evaluate outcomes among the 10% of simulations (200 simulations) with the highest sampled and lowest sampled values for each parameter.

Post TB Consequences	Parameter Value	Incremental Cost ($)	Incremental QALY	Incremental Death	Incremental Net Monetary Benefit	% Positive iNMB
Elevated Risk of Mortality	0th-10th percentile (95%UR)	18.5 million (3.7 to 34.4 million)	173 (110 to 240)	−22 (−28 to −16)	−1.2 million (−19.6 to 18.3 million)	47%
	90th-100th percentile (95%UR)	18.2 million (3.3 to 36.7 million)	192 (108 to 270)	−28 (−34 to −21)	1.0 million (−20.0 to 18.3 million)	59.5%
Persistent Morbidity	0th-10th percentile (95%UR)	18.4 million (3.5 to 36.8 million)	159 (106 to 217)	−26 (−33 to −18)	−2.5 million (−22.7 to 12.5 million)	43.5%
	90th-100th percentile (95%UR)	18.7 million (2.2 to 37.6 million)	220 (141 to 294)	−25 (−34 to −18)	3.3 million (−20.1 to 21.9 million)	66.5%
Increased Health Care Costs	0th-10th percentile (95%UR)	18.9 million (3.9 to 34.7 million)	180 (114 to 244)	−25 (−32 to −18)	−0.9 million (−18.4 to 17.7 million)	48.5%
	90th-100th percentile (95%UR)	17.7 million (2.2 to 36.7 million)	189 (118 to 267)	−25 (−34 to −17)	1.2 million (−18.2 to 19.0 million)	58.5%

Note: Incremental Net Monetary Benefit refers to the difference in net monetary benefit between the intervention strategy and the status quo
Abbreviations: TB = tuberculosis, UR = uncertainty range, iNMB = incremental net monetary benefit, QALY = quality adjusted life years

Appendix Table 5. Total quality adjusted life years lost due to tuberculosis when considering acute and long-term consequences in the status quo scenario with increased estimates for the acute consequences of TB

QALY Loss	Acute TB consequences only (Reference)	Post-TB consequence considered: elevated risk of mortality	Post-TB consequence considered: persistent morbidity	Both elevated mortality and persistent morbidity
Absolute QALY loss due to TB (95% UR)	375 (285 to 476)	554 (422 to 703)	545 (390 to 740)	720 (531 to 953)
Relative QALY loss due to TB compared to no post-TB consequences (95% UR)	1.0	1.5 (1.4 to 1.6)	1.5 (1.2 to 1.8)	1.9 (1.6 to 2.3)

Abbreviations: TB = tuberculosis, QALY = quality adjusted life year, UR = uncertainty range.

Appendix Table 6. Effects of post-tuberculosis consequences on incremental outcomes between intervention and status quo with increased estimates for the acute consequences of TB

Incremental outcomes between status quo and intervention	Acute TB consequence s only (Reference)	Post-TB consequence considered: elevated risk of mortality	Post-TB consequence considered: persistent morbidity	Post-TB consequence considered: increased healthcare costs	All post-TB consequences considered
Absolute incremental cost, $ (95% UR)	19.4 million (4.1 to 38.0 million)	19.4 million (4.1 to 38.0 million)	19.4 million (4.1 to 38.0 million)	18.7 million (3.3 to 37.3 million)	18.7 million (3.3 to 37.3 million)
Relative incremental cost ratio compared to no post-TB consequences (95% UR)	1.0	1.0	1.0	0.96 (0.86 to 0.99)	0.96 (0.86 to 0.99)
Absolute incremental QALY (95% UR)	100 (42 to 149)	156 (88 to 221)	149 (78 to 223)	100 (42 to 149)	203 (123 to 289)
Relative incremental QALY ratio compared to no post-TB consequences (95% UR)	1.0	1.7 (1.2 to 2.4)	1.6 (1.2 to 2.2)	1.0	2.3 (1.5 to 3.4)
Absolute incremental TB deaths (95% UR)	−12 (−15 to −8)	−25 (−33 to −18)	−12 (−15 to −8)	−12 (−15 to −8)	−25 (−33 to −18)
Relative incremental TB deaths ratio compared to no post-TB consequences (95% UR)	1.0	2.2 (1.6 to 3.0)	1.0	1.0	2.2 (1.6 to 3.0)
Incremental cost effectiveness ratio	$193,458	$124,186	$130,022	$187,084	$91,955
Percentage of ICERs under $50,000	3.4%	6.5%	6.5%	4%	13.8%
Percentage of ICERs under $100,000	11.9%	35.2%	31.6%	14.0%	60.2%
Percentage of ICERs under $150,000	33.1%	67.8%	64.3%	35.9%	86.6%

Note: Incremental outcomes refer to the difference between intervention and status quo per 400,000 individuals
Abbreviations: TB = tuberculosis, UR = uncertainty range, QALY = quality adjusted life year, UR = uncertainty range, ICER = Incremental Cost Effectiveness Ratio

Appendix Table 7. Effects of post-tuberculosis consequences on incremental outcomes between intervention and status quo when an interferon-gamma release assay is used instead of the tuberculin skin test

Incremental outcomes between status quo and intervention	Acute TB consequences only (Reference)	Post-TB consequence considered: elevated risk of mortality	Post-TB consequence considered: persistent morbidity	Post-TB consequence considered: increased healthcare costs	All post-TB consequences considered
Absolute incremental cost, $ (95% UR)	22.7 million (8.9 to 37.8 million)	22.7 million (8.9 to 37.8 million)	22.7 million (8.9 to 37.8 million)	22.0 million (8.4 to 37.0 million)	22.0 million (8.4 to 37.0 million)
Relative incremental cost ratio compared to no post-TB consequences (95% UR)	1.0	1.0	1.0	0.97 (0.92 to 0.99)	0.97 (0.92 to 0.99)
Absolute incremental QALY (95% UR)	82 (30 to 124)	138 (79 to 197)	131 (67 to 200)	82 (30 to 124)	186 (112 to 267)
Relative incremental QALY ratio compared to no post-TB consequences (95% UR)	1.0	1.6 (1.3 to 2.9)	1.6 (1.2 to 2.7)	1.0	2.2 (1.6 to 4.5)
Absolute incremental TB deaths (95% UR)	−12 (−15 to −7)	−25 (−33 to −18)	−12 (−15 to −7)	−12 (−15 to −7)	−25 (−33 to −18)
Relative incremental TB deaths ratio compared to no post-TB consequences (95% UR)	1.0	2.2 (1.6 to 3.0)	1.0	1.0	2.2 (1.6 to 3.0)
Incremental cost effectiveness ratio	$275,116	$163,984	$172,452	$267,343	$118,329
Percentage of ICERs under $50,000	0.9%	1.7%	1.7%	1.0%	4.7%
Percentage of ICERs under $100,000	2.9%	11.2%	10.4%	3.3%	33.2%
Percentage of ICERs under $150,000	8.9%	42.3%	36.0%	10.3%	74.6%

Note: Incremental outcomes refer to the difference between intervention and status quo per 400,000 individuals
Abbreviations: TB = tuberculosis, UR = uncertainty range, QALY = quality adjusted life year, UR = uncertainty range, ICER = Incremental Cost Effectiveness Ratio

Appendix Table 8. Effects of post-tuberculosis consequences on incremental outcomes between intervention and status quo with discount rate of 0%

Incremental outcomes between status quo and intervention	Acute TB consequences only (Reference)	Post-TB consequence considered: elevated risk of mortality	Post-TB consequence considered: persistent morbidity	Post-TB consequence considered: increased healthcare costs	All post-TB consequences considered
Absolute incremental cost, $ (95% UR)	18.1 million (1.1 to 37.0 million)	18.1 million (1.1 to 37.0 million)	18.1 million (1.1 to 37.0 million)	17.3 million (0.3 to 36.0 million)	17.3 million (0.3 to 36.0 million)
Relative incremental cost ratio compared to no post-TB consequences (95% UR)	1.0	1.0	1.0	0.93 (0.79 to 0.99)	0.93 (0.79 to 0.99)
Absolute incremental QALY (95% UR)	104 (37 to 156)	182 (106 to 257)	166 (83 to 251)	104 (37 to 156)	242 (146 to 343)
Relative incremental QALY ratio compared to no post-TB consequences (95% UR)	1.0	1.8 (1.3 to 3.1)	1.7 (1.2 to 2.6)	1.0	2.5 (1.6 to 4.5)
Absolute incremental TB deaths (95% UR)	−14 (−18 to −8)	−32 (−42 to −22)	−14 (−18 to −8)	−14 (−18 to −8)	−32 (−42 to −22)
Relative incremental TB deaths ratio compared to no post-TB consequences (95% UR)	1.0	2.4 (1.7 to 3.3)	1.0	1.0	2.4 (1.7 to 3.3)
Incremental cost effectiveness ratio	$173,586	$99,616	$109,123	$166,260	$71,714
Percentage of ICERs under $50,000	5.7%	13%	11.4%	7.2%	27.1%
Percentage of ICERs under $100,000	18.4%	53.8%	45.4%	21.3%	75.6%
Percentage of ICERs under $150,000	42.5%	81%	73.6%	45.6%	94.3%

Note: Incremental outcomes refer to the difference between intervention and status quo per 400,000 individuals
Abbreviations: TB = tuberculosis, UR = uncertainty range, QALY = quality adjusted life year, UR = uncertainty range, ICER = Incremental Cost Effectiveness Ratio

Appendix Table 9. Effects of post-tuberculosis consequences on incremental outcomes between intervention and status quo with discount rate of 3%

Incremental outcomes between status quo and intervention	Acute TB consequences only (Reference)	Post-TB consequence considered: elevated risk of mortality	Post-TB consequence considered: persistent morbidity	Post-TB consequence considered: increased healthcare costs	All post-TB consequences considered
Absolute incremental cost, $ (95% UR)	20.4 million (6.0 to 38.7 million)	20.4 million (6.0 to 38.7 million)	20.4 million (6.0 to 38.7 million)	19.8 million (5.4 to 38.1 million)	19.8 million (5.4 to 38.1 million)
Relative incremental cost ratio compared to no post-TB consequences (95% UR)	1.0	1.0	1.0	0.97 (0.91 to 0.99)	0.97 (0.91 to 0.99)
Absolute incremental QALY (95% UR)	66 (21 to 100)	106 (55 to 154)	105 (51 to 160)	66 (21 to 100)	144 (82 to 209)
Relative incremental QALY ratio compared to no post-TB consequences (95% UR)	1.0	1.6 (1.2 to 2.8)	1.6 (1.2 to 2.7)	1.0	2.2 (1.5 to 4.2)
Absolute incremental TB deaths (95% UR)	−10 (−13 to −6)	−20 (−26 to −14)	−10 (−13 to −6)	−10 (−13 to −6)	−20 (−26 to −14)
Relative incremental TB deaths ratio compared to no post-TB consequences (95% UR)	1.0	2.0 (1.5 to 2.8)	1.0	1.0	2.0 (1.5 to 2.8)
Incremental cost effectiveness ratio	$309,964	$192,188	$194,262	$301,767	$137,407
Percentage of ICERs under $50,000	1.1%	2.1%	2.3%	1.3%	4.8%
Percentage of ICERs under $100,000	3.2%	9.7%	10.2%	3.8%	27.2%
Percentage of ICERs under $150,000	9.3%	32.3%	31.5%	10.5%	61.3%

Note: Incremental outcomes refer to the difference between intervention and status quo per 400,000 individuals
Abbreviations: TB = tuberculosis, UR = uncertainty range, QALY = quality adjusted life year, UR = uncertainty range, ICER = Incremental Cost Effectiveness Ratio

Appendix Table 10. Total quality adjusted life years lost due to tuberculosis when considering acute and long-term consequences in the status quo scenario with decreased estimates for the post-TB elevated risk of mortality

QALY loss	Acute TB consequences only (Reference)	Post-TB consequence considered: elevated risk of mortality	Post-TB consequence considered: persistent morbidity	Both elevated mortality and persistent morbidity
Absolute QALY loss due to TB (95% UR)	312 (242 to 386)	326 (251 to 407)	482 (338 to 664)	496 (346 to 686)
Relative QALY loss due to TB compared to no post-TB consequences (95% UR)	1.0	1.05 (1.02 to 1.09)	1.5 (1.2 to 2.0)	1.6 (1.3 to 2.0)

Abbreviations: TB = tuberculosis, QALY = quality adjusted life year, UR = uncertainty range.

Appendix Table 11. Effects of post-tuberculosis consequences on incremental outcomes between intervention and status quo with decreased estimates for the post-TB elevated risk of mortality

Incremental outcomes between status quo and intervention	Acute TB consequences only (Reference)	Post-TB consequence considered: elevated risk of mortality	Post-TB consequence considered: persistent morbidity	Post-TB consequence considered: increased healthcare costs	All post-TB consequences considered
Absolute incremental cost, $ (95% UR)	19.4 million (4.1 to 38.1 million)	19.4 million (4.1 to 38.1 million)	19.4 million (4.1 to 38.1 million)	18.7 million (3.3 to 37.5 million)	18.7 million (3.3 to 37.5 million)
Relative incremental cost ratio compared to no post-TB consequences (95% UR)	1.0	1.0	1.0	0.97 (0.87 to 0.99)	0.97 (0.87 to 0.99)
Absolute incremental QALY (95% UR)	82 (29 to 124)	89 (35 to 130)	131 (66 to 200)	82 (29 to 124)	137 (71 to 206)
Relative incremental QALY ratio compared to no post-TB consequences (95% UR)	1.0	1.1 (1.0 to 1.2)	1.7 (1.2 to 2.6)	1.0	1.8 (1.3 to 2.8)
Absolute incremental TB deaths (95% UR)	−12 (−15 to −7)	−14 (−18 to −10)	−12 (−15 to −7)	−12 (−15 to −7)	−14 (−18 to −10)
Relative incremental TB deaths ratio compared to no post-TB consequences (95% UR)	1.0	1.2 (1.1 to 1.3)	1.0	1.0	1.2 (1.1 to 1.3)
Incremental cost effectiveness ratio	$234,806	$218,601	$147,662	$227,067	$136,556
Percentage of ICERs under $50,000	2.8%	2.9%	5.4%	3.2%	6.6%
Percentage of ICERs under $100,000	8.3%	9.1%	24.5%	9.2%	28.9%
Percentage of ICERs under $150,000	21.1%	25.3%	54.3%	24.1%	60.0%

Note: Incremental outcomes refer to the difference between intervention and status quo per 400,000 individuals
Abbreviations: TB = tuberculosis, UR = uncertainty range, QALY = quality adjusted life year, UR = uncertainty range, ICER = Incremental Cost Effectiveness Ratio

Appendix Table 12. Total quality adjusted life years lost due to tuberculosis when considering acute and long-term consequences in the status quo scenario with decreased estimates for the TB progression rate

QALY loss	Acute TB consequences only (Reference)	Post-TB consequence considered: elevated risk of mortality	Post-TB consequence considered: persistent morbidity	Both elevated mortality and persistent morbidity
Absolute QALY loss due to TB (95% UR)	275 (210 to 349)	428 (318 to 554)	425 (292 to 603)	575 (411 to 784)
Relative QALY loss due to TB compared to no post-TB consequences (95% UR)	1.0	1.6 (1.4 to 1.7)	1.5 (1.2 to 2.0)	2.1 (1.7 to 2.5)

Abbreviations: TB = tuberculosis, QALY = quality adjusted life year, UR = uncertainty range.

Appendix Table 13. Effects of post-tuberculosis consequences on incremental outcomes between intervention and status quo with decreased estimates for the TB progression rate

Incremental outcomes between status quo and intervention	Acute TB consequences only (Reference)	Post-TB consequence considered: elevated risk of mortality	Post-TB consequence considered: persistent morbidity	Post-TB consequence considered: increased healthcare costs	All post-TB consequences considered
Absolute incremental cost, $ (95% UR)	20.3 million (6.0 to 38.6 million)	20.3 million (6.0 to 38.6 million)	20.3 million (6.0 to 38.6 million)	19.7 million (5.3 to 38.0 million)	19.7 million (5.3 to 38.0 million)
Relative incremental cost ratio compared to no post-TB consequences (95% UR)	1.0	1.0	1.0	0.97 (0.90 to 0.99)	0.97 (0.90 to 0.99)
Absolute incremental QALY (95% UR)	71 (14 to 120)	125 (59 to 186)	113 (47 to 186)	71 (14 to 120)	165 (91 to 251)
Relative incremental QALY ratio compared to no post-TB consequences (95% UR)	1.0	1.7 (1.3 to 3.3)	1.6 (1.2 to 2.9)	1.0	2.3 (1.6 to 5.0)
Absolute incremental TB deaths (95% UR)	−10 (−14 to −6)	−24 (−31 to −16)	−10 (−14 to −6)	−10 (−14 to −6)	−24 (−31 to −16)
Relative incremental TB deaths ratio compared to no post-TB consequences (95% UR)	1.0	2.4 (1.7 to 3.5)	1.0	1.0	2.4 (1.7 to 3.5)
Incremental cost effectiveness ratio	$286,499	$162,481	$180,016	$278,609	$119,095
Percentage of ICERs under $50,000	1.5%	3.3%	2.9%	1.8%	6.8%
Percentage of ICERs under $100,000	4.7%	16.6%	14.6%	5.6%	38.3%
Percentage of ICERs under $150,000	13.2%	46.6%	37.4%	14.5%	70%

Note: Incremental outcomes refer to the difference between intervention and status quo per 400,000 individuals
Abbreviations: TB = tuberculosis, UR = uncertainty range, QALY = quality adjusted life year, UR = uncertainty range, ICER = Incremental Cost Effectiveness Ratio

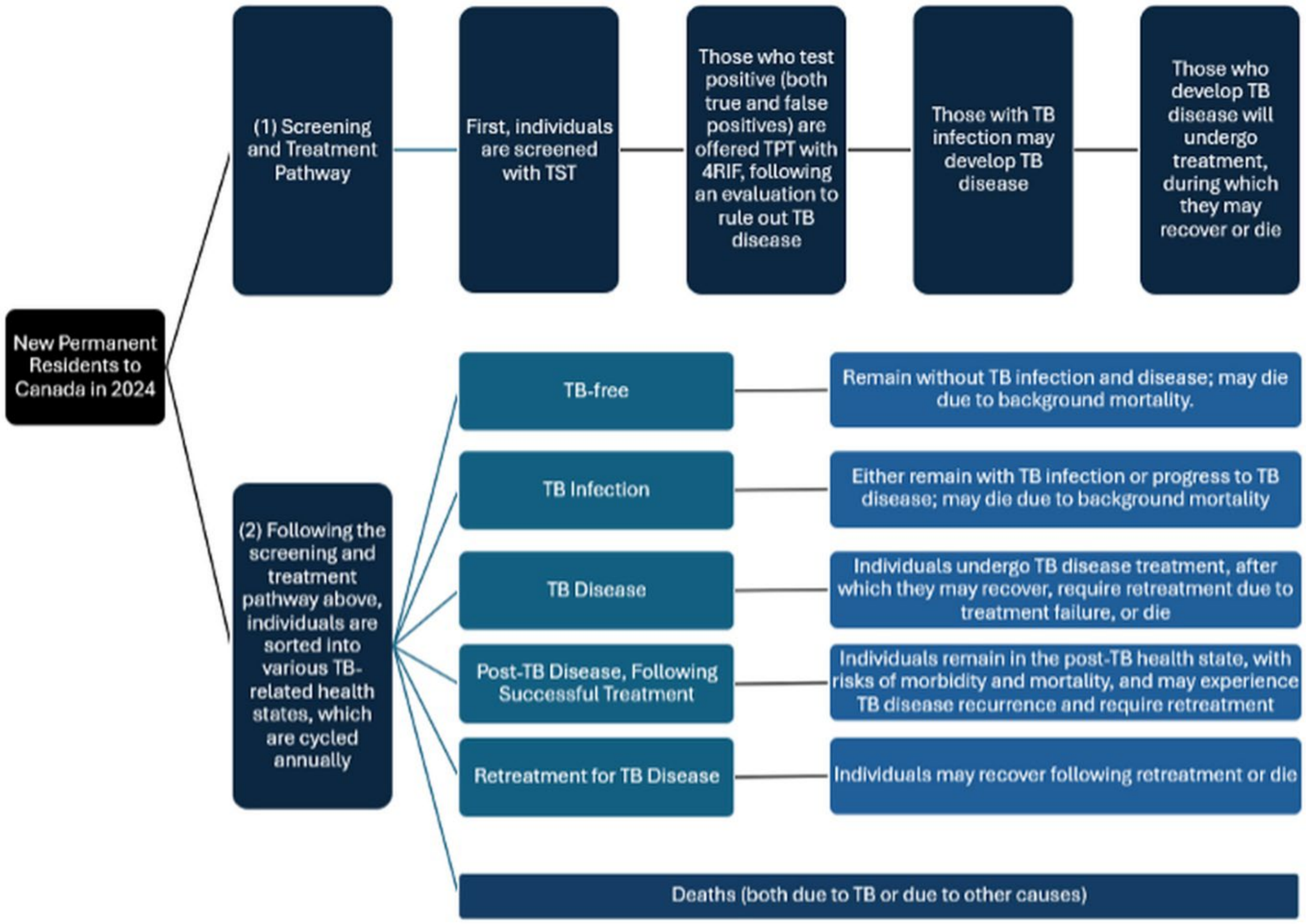

Appendix Figure 1. Simplified model structure. Abbreviations: TB = tuberculosis; TST = tuberculin skin test; TPT = tuberculosis preventive treatment; 4RIF = 4 months of daily rifampin

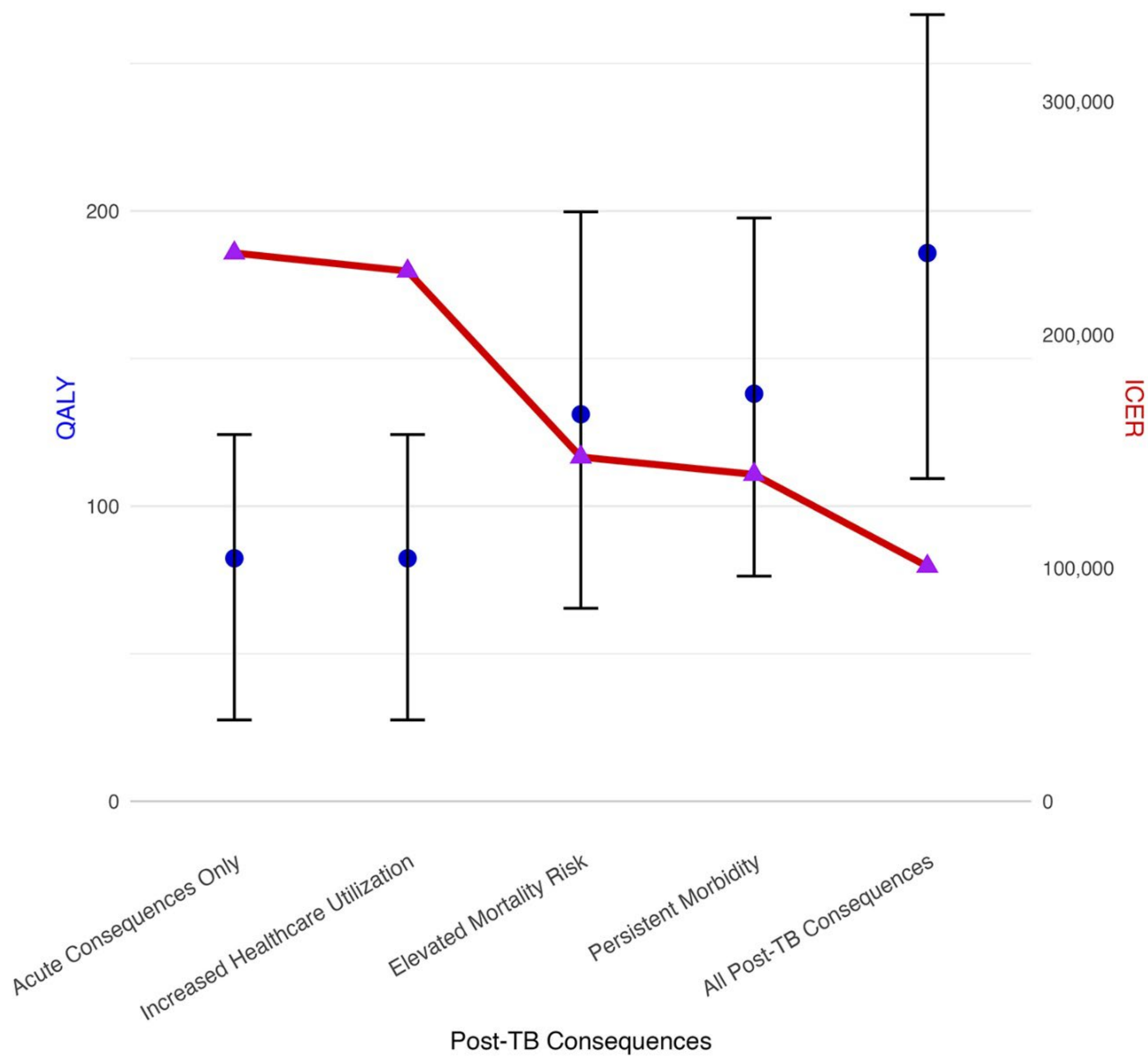

Appendix Figure 2. Effect of post-TB consequences on quality adjusted life years (blue dots) and incremental cost effectiveness ratio (red line). Note: Error bars represent the 95% uncertainty range around estimated incremental QALYs. Abbreviations: QALY = quality adjusted life year, ICER = Incremental Cost Effectiveness Ratio, TB = tuberculosis

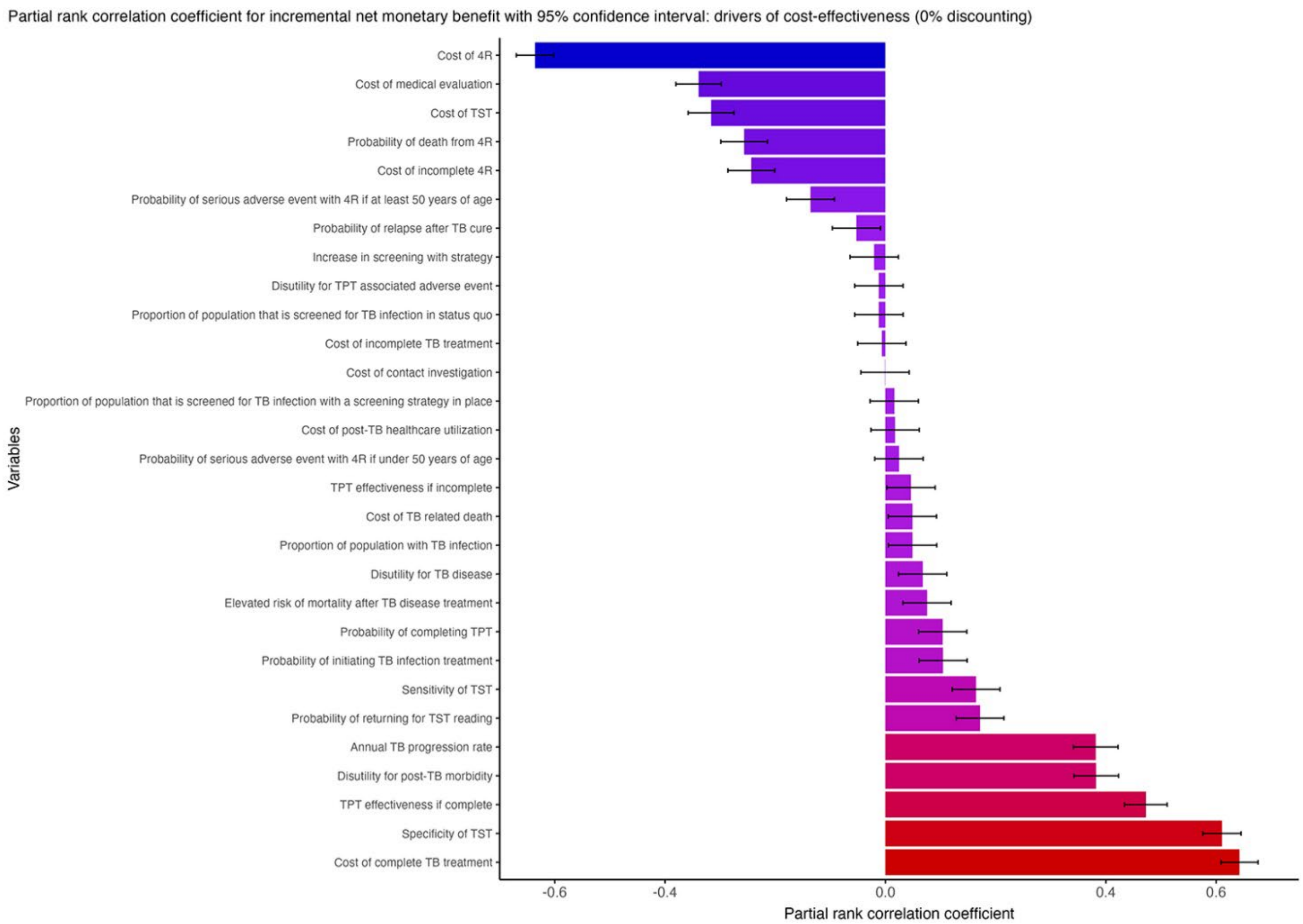

Appendix Figure 3. Partial rank correlation coefficient for incremental net monetary benefit: drivers of cost-effectiveness (0% discounting). Note: Incremental Net Monetary Benefit refers to the difference in net monetary benefit between the intervention and the status quo. In this analysis, parameters with more extreme positive PRCC estimates improve the incremental net monetary benefit as they increase (e.g., as specificity of TST increases, so too does the estimated incremental net monetary benefit). Parameters with more extreme negative PRCC estimates worsen the incremental net monetary benefit as they increase (e.g., as the probability of death from the tuberculosis preventive treatment regimen 4 months of rifampin increases, the estimated incremental net monetary benefit decreases). Abbreviations: TB = tuberculosis, 4R = 4 months of rifampin, TPT = tuberculosis preventative treatment, TST = tuberculin skin test, PRCC = partial rank correlation coefficient

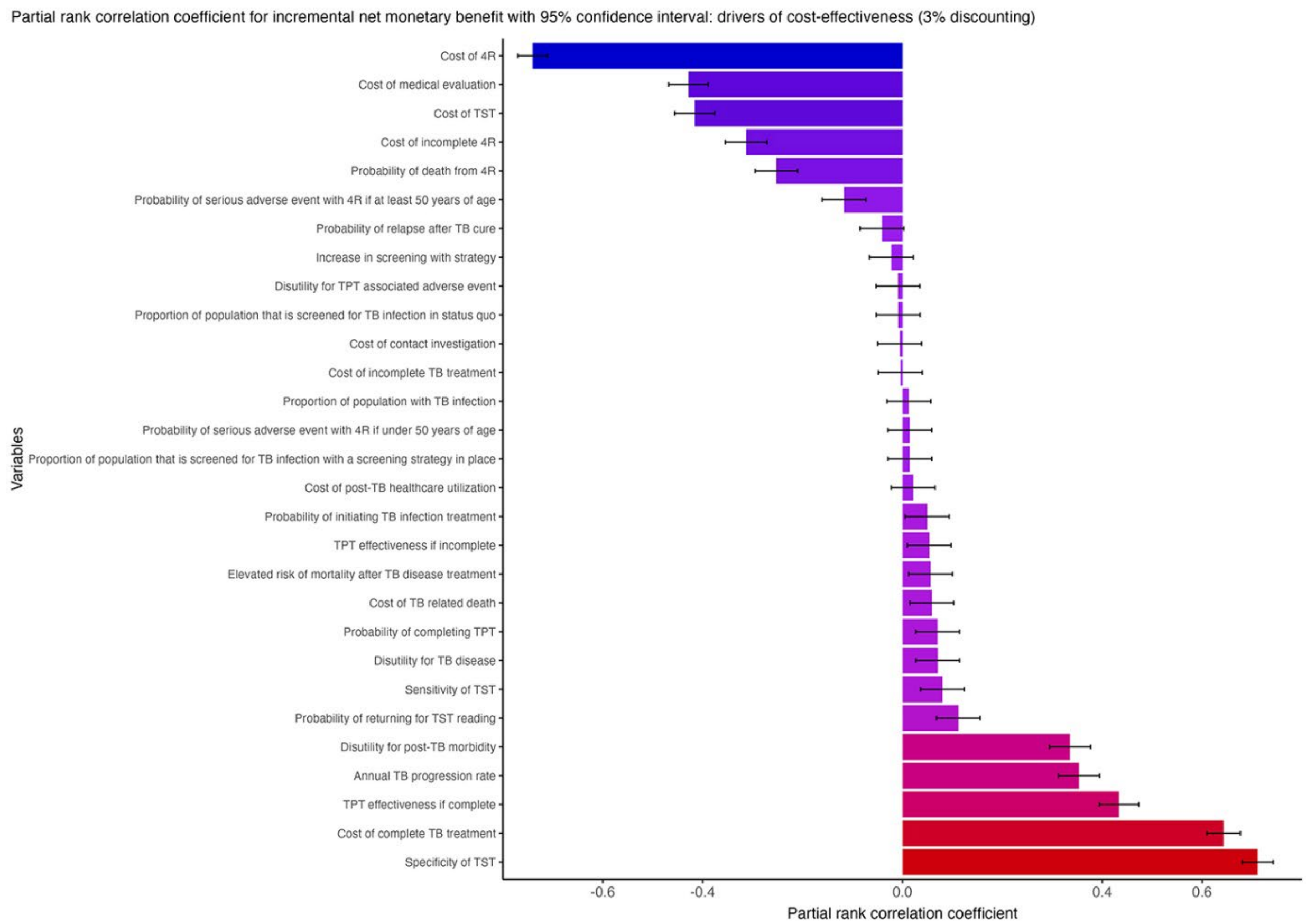

Appendix Figure 4. Partial rank correlation coefficient for incremental net monetary benefit: drivers of cost-effectiveness (3% discounting). Note: Incremental Net Monetary Benefit refers to the difference in net monetary benefit between the intervention and the status quo. In this analysis, parameters with more extreme positive PRCC estimates improve the incremental net monetary benefit as they increase (e.g., as specificity of TST increases, so too does the estimated incremental net monetary benefit). Parameters with more extreme negative PRCC estimates worsen the incremental net monetary benefit as they increase (e.g., as the probability of death from the tuberculosis preventive treatment regimen 4 months of rifampin increases, the estimated incremental net monetary benefit decreases). Abbreviations:
TB = tuberculosis, 4R = 4 months of rifampin, TPT = tuberculosis preventative treatment,
TST = tuberculin skin test, PRCC = partial rank correlation coefficient
